# Modal gain characteristics of a two-section InGaAs/GaAs double quantum well passively mode-locked laser with asymmetric waveguide

**DOI:** 10.1038/s41598-022-09136-6

**Published:** 2022-03-23

**Authors:** Zhongliang Qiao, Xiang Li, Jia Xu Brian Sia, Wanjun Wang, Hong Wang, Zaijin Li, Zhibin Zhao, Lin Li, Xin Gao, Baoxue Bo, Yi Qu, Guojin Liu, Chongyang Liu

**Affiliations:** 1 Hainan Province Key Laboratory of Laser Technology and Photoelectric Functional Materials, School of Physics and Electronic Engineering, Hainan Normal University, No. 99 Longkun Road, Haikou, 571158 Hainan China; 2 Temasek Laboratories@NTU (TL@NTU), Nanyang Technological University, 50 Nanyang Drive, Singapore, 637553 Singapore; 3 School of Electrical and Electronic Engineering, Nanyang Technological University, Singapore, 639798 Singapore; 4 National Key Laboratory On High Power Semiconductor Lasers, Changchun University of Science and Technology, No. 7186 Weixing Road, Changchun, 130022 Jilin China

**Keywords:** Engineering, Materials science, Optics and photonics, Physics

## Abstract

Monolithic two-section InGaAs/GaAs double quantum well (DQW) passively mode-locked lasers (MLLs) with asymmetric waveguide, consisting of the layers of p-doped AlGaAs waveguide and no-doped InGaAsP waveguide, emitting at ~ 1.06 μm, with a fundamental repetition rate at ~ 19.56 GHz have been demonstrated. Modal gain characteristics, such as a gain bandwidth and a gain peak wavelength of the MLL, as a function of the saturable absorber (SA) bias voltage (*V*_*a*_) as well as the injection current of gain section (*I*_g_), were investigated by the Hakki-Paoli method. With the increase of *V*_*a*_, the lasing wavelength and net modal gain peak of the MLL both exhibited red-shifts to longer wavelength significantly, while the modal gain bandwidth was narrowed. Both the net modal gain bandwidth and gain peak of the MLL followed a polynomial distribution versus the reverse bias at the absorber section. In addition, for the first time, it was found that *V*_*a*_ had an obvious effect on the modal gain characteristics of the MLL.

## Introduction

Monolithic semiconductor passively mode-locked lasers (MLLs) are ideal light sources for short optical pulses, and are particularly attractive for their low cost, small size and ease of pumping. In recent years, light sources emitting at 1.06 µm, with an ultra-short pulse, have attracted great research interests, which are promising for many applications such as biometric imaging^1,2^, frequency comb^3^, two-photon microscopy (TPM)^4^, lidar remote sensing^5,6^, and so on. In addition, they are also used as pump sources^7,8^. These are of particular interest given their substantial impact on the applications in photonics and system integration.

Multi-section waveguide structure is generally adopted in a semiconductor mode-locked laser^9–12^, where the gain section is forward biased and saturable absorber (SA) section is reversely biased. The gain properties of single-section different material quantum well (QW)/quantum dot (QD) lasers also have been intensively studied. For increasing the output power in passively mode locked semiconductor lasers, two GaAs/AlGaAs epilayer designs, a double QW design operating at 830 nm and a single QW design operating at 795 nm, were compared, with vertical mode sizes of 0.5 and 0.75 μm, respectively, and a very high peak was demonstrated^11^. High-power QD broad area lasers emitting at 980 nm are presented with continuous-wave output powers of 4.3 W from a 50 μm stripe width laser and of 6.3 W from a 100 μm stripe width laser were achieved at 15 °C^13^. Amplified spontaneous emission (ASE) measurements of a quantum-well coupled quantum-dot (QW–QD) laser are investigated in the QW–QD laser consists of an auxiliary QW which assists in carrier collection while tunneling of carriers takes place from the well to the dot region, revealing a low linewidth enhancement factor over a flat spectrum for these GaAs–InGaAs–InAs QW–QD lasers^14^. Moreover, QD structures emit ultraviolet (UV) wavelength by varying the boron mole fraction in the QD active region or the barrier, the total polarization of which is decreasing for the Al-containing systems^15^.The high net modal gain was obtained when the waveguide Fermi energy was considered which meant that the increment comes from the material gain, not from the confinement factor^16^. DQW MLLs have higher output power and Electro-optic conversion efficiency than QD MLLs, and multi-section DQW MLLs also can generate shorter pulse^17^. However, the modal gain characteristics, i.e., modal gain bandwidth, peak, of multi-section InGaAs/GaAs DQW MLLs remains unexplored. All these have an important impact on the application of MLLs. Furthermore, it is well known that the thermal stability of InGaAsP semiconductor laser is even better than that of AlGaAs by reducing Auger recombination^18^, p-doped AlGaAs is easier to achieve high-quality growth than p-doped InGaAsP materials, and the ultrafast carrier dynamics in InGaAsP is better than that in AlGaAs, these characteristics of which is more suitable for generating shorter light pulses in an electrically pumped edge emitting semiconductor MLL^11,12,18^.

We report and demonstrate shorter pulse width and higher output power in a monolithic two-section passive DQW MLL with asymmetric waveguide emitting at 1.06 μm, and the modal gain characteristics is investigated by the Hakki-Paoli method^19–23^ for better understanding on its bias-dependent output. The layers of p-doped AlGaAs waveguide and un-doped InGaAsP waveguide are included in the asymmetric waveguide. For the first time to our knowledge, picosecond pulse generation was achieved by a two-section InGaAs/GaAs DQW MLL with asymmetric waveguide emitting at ~ 1.06 μm without external pulse compression, and the radio frequency (RF) spectrum, dynamic spectrum, net modal gain, and photocuurent performances on the absorber are systematically analyzed for two-section InGaAs/GaAs DQW MLLs. The pulse width and the peak power are also measured for the fabricated monolithic two-section MLL.

## Device overview and experimental setup

### Material structure

In this work, 1.06-µm InGaAs/GaAs DQW laser structures, with asymmetric waveguide, were grown on n-GaAs (100) substrates by metalorganic chemical vapor deposition (MOCVD). The detailed epitaxial structure is shown in the Table 1, similar to our previous study^24^.

**Table 1:** 1.06-µm InGaAs dqw laser with asymmetric heterostructure layers.

Material	Thickness (nm)	Doping (cm^-3^)
GaAs	250	p, ˃2e19
Al_0.55_Ga_0.45_As	300	p, 6e18
Al_0.35_Ga_0.65_As	300	p, 8e17
Al_0.3_Ga_0.7_As	300	p, 8e17
Al_0.05_Ga_0.95_As	200	p, 8e16
GaAs	8	Barrier
In_0.2_Ga_0.8_As	7	Quantum well
GaAs	8	Barrier
In_0.2_Ga_0.8_As	7	Quantum well
GaAs	8	Barrier
In_0.03_Ga_0.97_As_0.95_P_0.05_	200	Non-doped
In_0.32_Ga_0.68_As_0.4_P_0.6_	300	n, 8e16
In_1−x_Ga_x_As_0.5_P_0.5_	100	n, 8e17
In_1−x_Ga_x_As_0.7_P_0.3_	100	n, 8e17
In_1−x_Ga_x_As_0.9_P_0.1_	800	n, 8e17
GaAs	200	n, 2e18

### Device structure and manufacturing process

**Figure 1:**
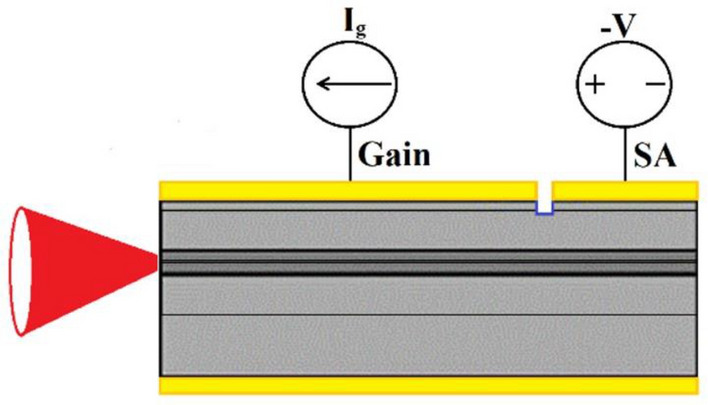
Sketch (side view) of the two-section MLL.

The MLL fabrication process is similar to those reported in Refs.^25,26^. First, a 6 µm-wide and 1.3 µm-deep ridge waveguide was formed by standard lithography and wet etching. A 300-nm-thick SiO_2_ film was deposited as an electrical isolation layer, and a 3 µm-wide p-type electrode window was opened on the etched ridge less than 6 µm-wide (due to the ridge etch). Then, a 10 µm-wide electrical isolation region was patterned by another lithography step. Ti/Au layers were evaporated to form the p-side ohmic contact. After that, lift-off process was carried out to expose the electrical isolation region, and a consequent wet etching process was used to improve the electrical isolation effect. An isolation resistance of ~ 1 kΩ was achieved between the two sections. Finally, the substrate was thinned, Ni/Ge/Au/Ni/Au layers were evaporated on the n-side, and then an ohmic contact layer was formed after 410 °C annealing. The processed wafer was cleaved into laser chips for device characterization. During the characterization, a thermo-electrical cooler (TEC) was used to control the operation temperature for the non-soldered MLL. In this study, the MLL was tested p-side up on the TEC, the length of the gain section (*L*_g_) and the saturable absorber (SA) section (*L*_*a*_) are 1576 µm and 404 µm, respectively. The gain section is forward driven by current (*I*_g_) while the absorber section is reversely biased with *V*_a_. The side view of the two-section MLL with gain section and SA section is shown in Fig. 1. No coating on the cavity facet of MLLs was prepared.

### Measurement setup

The reverse bias voltage is directly applied to the saturation absorption section by Keithley 2401 power supply, and the photocurrent value is automatically read.

**Figure 2:**
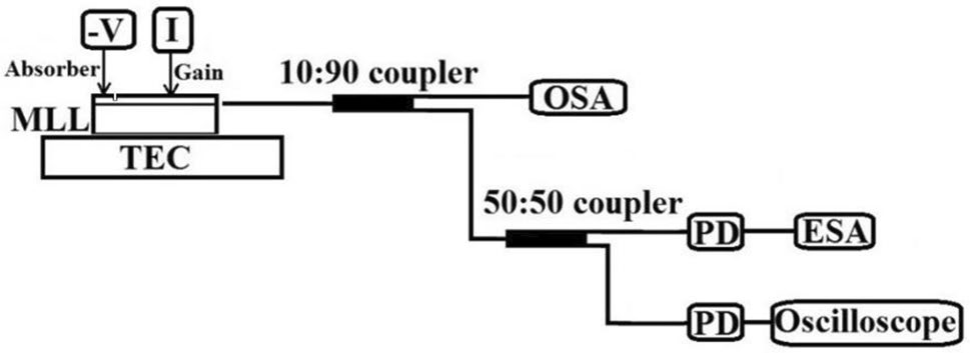
Schematic diagram of the MLL measurement setup.

The output light from the gain section facet was first coupled into a single mode fiber, then was split by a 10:90 fiber coupler, the 10 percent was guided into an optical spectrum analyzer (OSA, AQ6370C), and the 90 percent was further split into two equal parts: one part was fed into a 50-GHz high-speed photodiode (PD) monitored by a 30-GHz real-time oscilloscope (DSO93004L); another part was fed as the signal source for an electrical spectrum analyzer (ESA, N9030A). The details are shown in the Fig. 2.

### Experimental results and analysis

**Figure 3:**
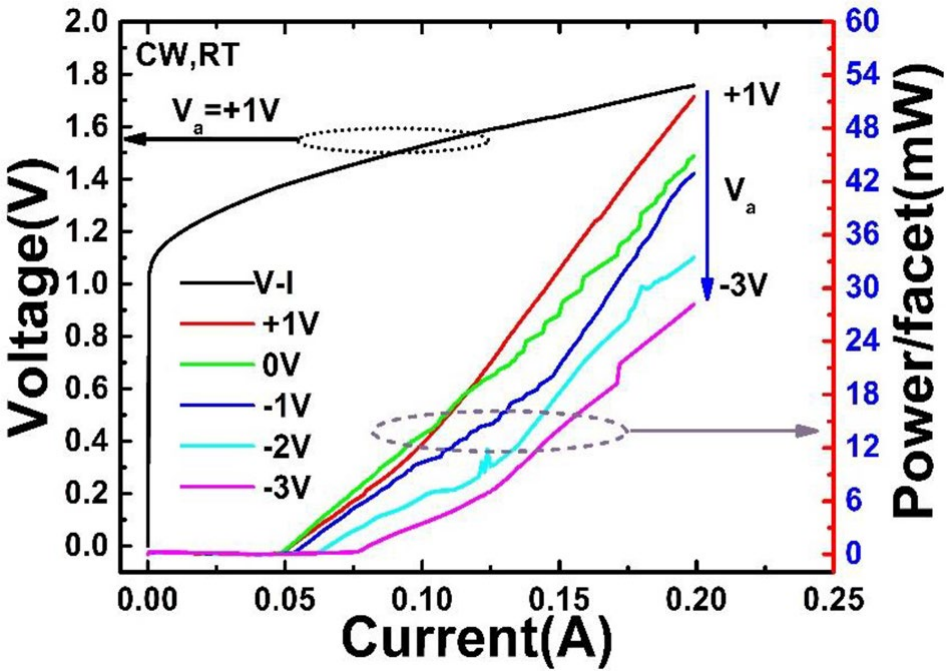
*L-I* and *V-I* curves of the laser with the absorber section bias (*V*_a_) varied from + 1 to − 3 V at room temperature.

The measurement sensitivity is set constant for all measurements. For the tested laser in this study, the wet etched ridge width provides single spatial mode operation condition while operating in the mode locking regime. Figure 3 shows the light output power characteristics under different reverse bias voltages of the absorber section for the tested device in the continuous wave (CW) operation at room temperature (RT). The bias voltage (*V*_*a*_) applied to the absorber section varied from + 1 to − 3 V, with a 1 V step, and *I*_g_ increased from 0 to 200 mA under each *V*_a_. The use of long absorber sections (up to 20.4% of total length) resulted in increased absorption, and was beneficial to mode locking and obtains ultrashort pulse. As the negative bias on the absorption region increases, the threshold current changes obviously differently in two intervals, one is the interval from + 1 to − 1 V, where the threshold current increases slowly; the other is the interval from − 1 to − 3 V, the threshold current increases rapidly with increased negative bias. This phenomenon should be caused by the strong absorption of the absorption section under a strong negative bias *V*_a_. The L-I and V-I curves show the good operating characteristics of the MLL. The increase of the reverse bias voltage leads to the narrowing of the band gap of the saturable absorption region. The narrowed band gap increases the absorption of the passing photons. The absorbed photons are converted into electrons, and a large number of electrons are generated to form a photocurrent. The increase of the photocurrent makes the temperature of the quantum well region, which further leads to a narrowing of the band gap of the DQW^26^.

**Figure 4:**
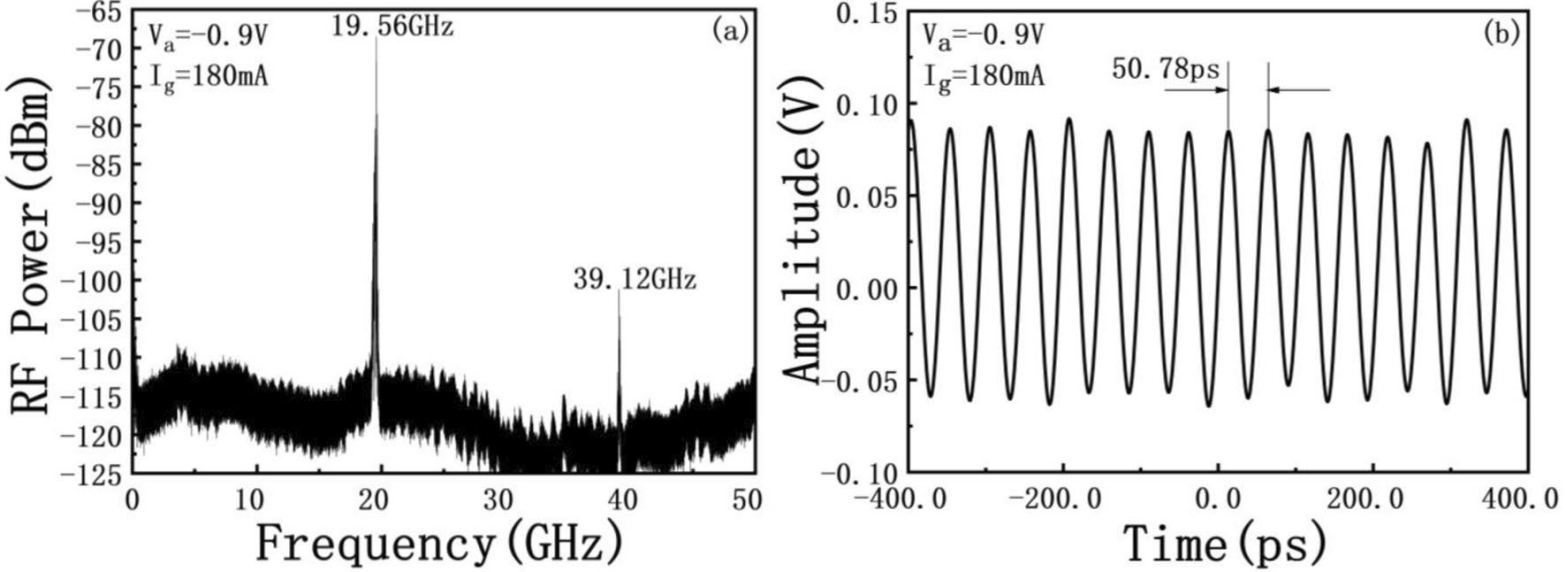
(**a**) Frequency spectrum of the MLL under the bias condition (*I*_g_ = 180 mA, *V*_a_ =  − 0.9 V). (**b**) Pulse trains under the same bias condition.

**Figure 5:**
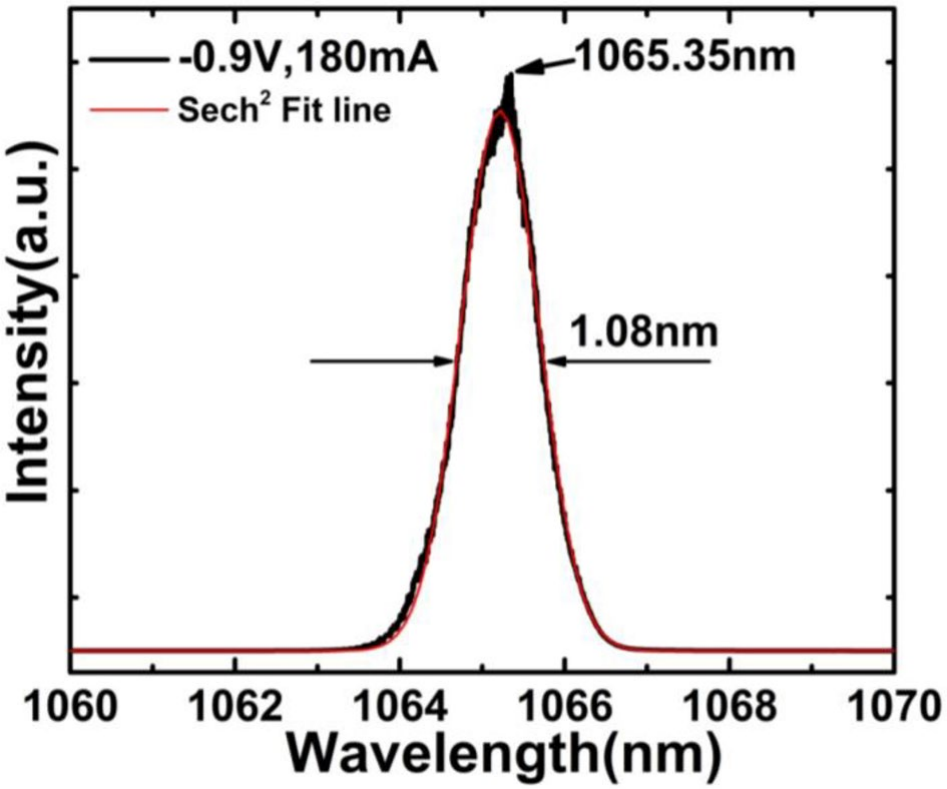
Spectrum of the MLLs when PW = 1.1 ps.

Stable mode locking was achieved over a wide range of *V*_*a*_ (from 0 to − 3 V). The RF spectra were measured at *I*_g_ = 180 mA and *V*_a_ =  − 0.9 V as shown in Fig. 4a using a resolution bandwidth (RBW) of 10 kHz. The fundamental repetition rate with more than 42 dB signal to noise ratio (SNR) at room temperature is ~ 19.56 GHz, corresponding to the photon round-trip time in the 1.99 mm-long laser cavity. The second harmonic frequency at ~ 39.12 GHz is also marked in the figure with ~ 15 dB SNR, which indicate an efficient mode locking. The RF signal from the photodetector was also fed into a 30 GHz real-time oscilloscope (DSO93004L). Figure 4b shows the pulse train observed on the oscilloscope. The time interval between two pulses is ~ 50.78 ps, corresponding well to a repetition rate at ~ 19.69 GHz, which is very close to the fundamental repetition rate ~ 19.56 GHz. The response resolution of the real-time oscilloscope in picosecond and femtosecond level is not enough. Therefore, the measured time pulse train is actually an envelope of ultra-narrow pulses. It can also be seen from Fig. 4b that the distribution of the time-domain pulse train presents a more uniform and regular time distribution and the cycle time change is very small and barely noticeable, which shows that the time-domain pulse envelope is much closer to no chirp. The obvious chirp effect is usually reflected in the jitter of the time domain signal and the difference between the spectral signal and the hyperbolic secant fit. The more obvious the jitter and periodic instability of the time domain signal, the chirp is stronger, and vice versa. On the other hand, the greater difference between the spectral signal and its hyperbolic secant fitting, which indicates a stronger chirp effect. In the Figs. 4b and 5, for our test results, it indirectly reflects the weak chirp effect. This provides a basis for calculating its ultra-narrow pulse width using the ideal sech^2^ shaped of the time-bandwidth product (TBP) later.

The full width at half maximum (FWHM) of the emission spectrum (Δ*λ*) is ~ 1.08 nm, and the optical spectrum of the laser is centered at 1065.35 nm. The tested peak and fitted peak curves are showed in Fig. 5. The ideal Lorentz-shaped TBP of ~ 0.22 is a smaller value than the ideal sech^2^ shaped pulses TBP of ~ 0.315. Here, taking into account that it is not significantly affected by the chirp, and assuming an ideal Fourier transform-limited pulse, a pulse width (PW) of ~ 1.1 ps can be expected by the sech^2^ shaped. Under this condition, the average power from the MLL is ~ 71 mW, corresponding to a peak power ~ 3.3 W. To our knowledge, this is the narrowest pulse width and the highest peak output power obtained from an electrically pumped edge emitting GaAs-based two-section semiconductor DQW-MLL. It can be seen that pulses get broadened with increasing *I*_g_, and narrowed with increasing *V*_a_.

**Figure 6:**
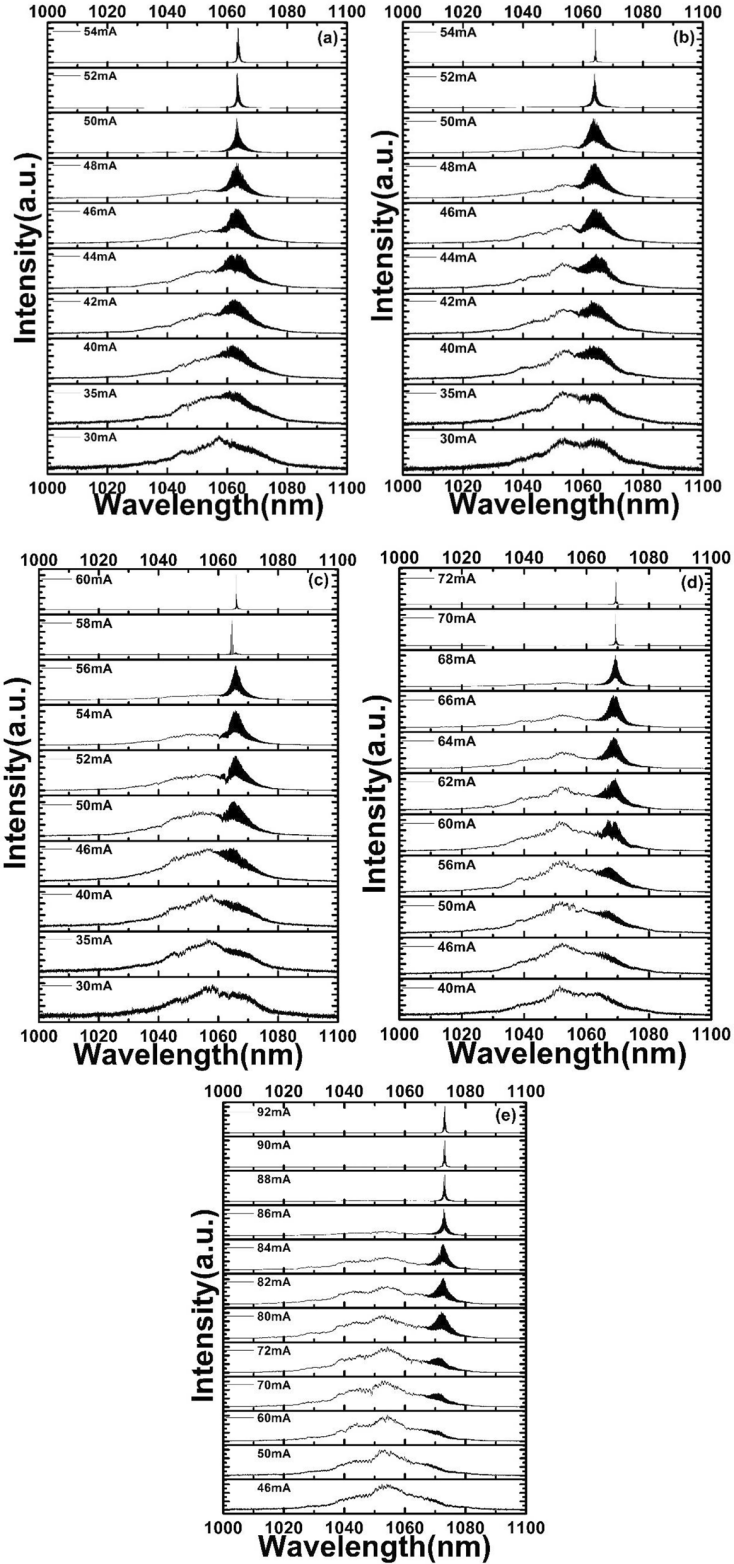
The spectra measured from the InGaAs/GaAs DQW MLL at different *V*_*a*_ (**a** *V*_*a*_ =  + 1 V; **b** *V*_*a*_ = 0 V; **c** *V*_*a*_ =  − 1 V; **d** *V*_*a*_ =  − 2 V; **e** *V*_*a*_ =  − 3 V).

Figure 6 shows the electroluminescence spectrum measured at different *V*_a_ (a: *V*_*a*_ =  + 1 V ; b: *V*_*a*_ = 0 V ; c: *V*_*a*_ =  − 1 V ; d: *V*_*a*_ =  − 2 V ; e: *V*_*a*_ =  − 3 V). It can be found that the redshift of the lasing peak occurred is moved further away from the center of the spontaneous emission spectrum with an increasing reverse bias. As the reverse bias *V*_a_ increases, the band-gap of the absorption region shrinks significantly due to the quantum confined Stark effect (QCSE). In the gain spectrum of the MLL, lasing tends to happen at long wavelength while mode gain reaches the threshold current^26^.

The peak is shifted from 1063 to 1074 nm near the threshold current under different *V*_a_. The peaks redshift, and become narrower and higher with *I*_g_ under increasing bias *V*_a_ in Fig. 6a–e. Especially, it is also found that lasing peak will be getting obviously narrower as reverse bias changes from + 1 to − 3 V. This should be caused by the more bandgap shrinkage under big reverse bias.

**Figure 7:**
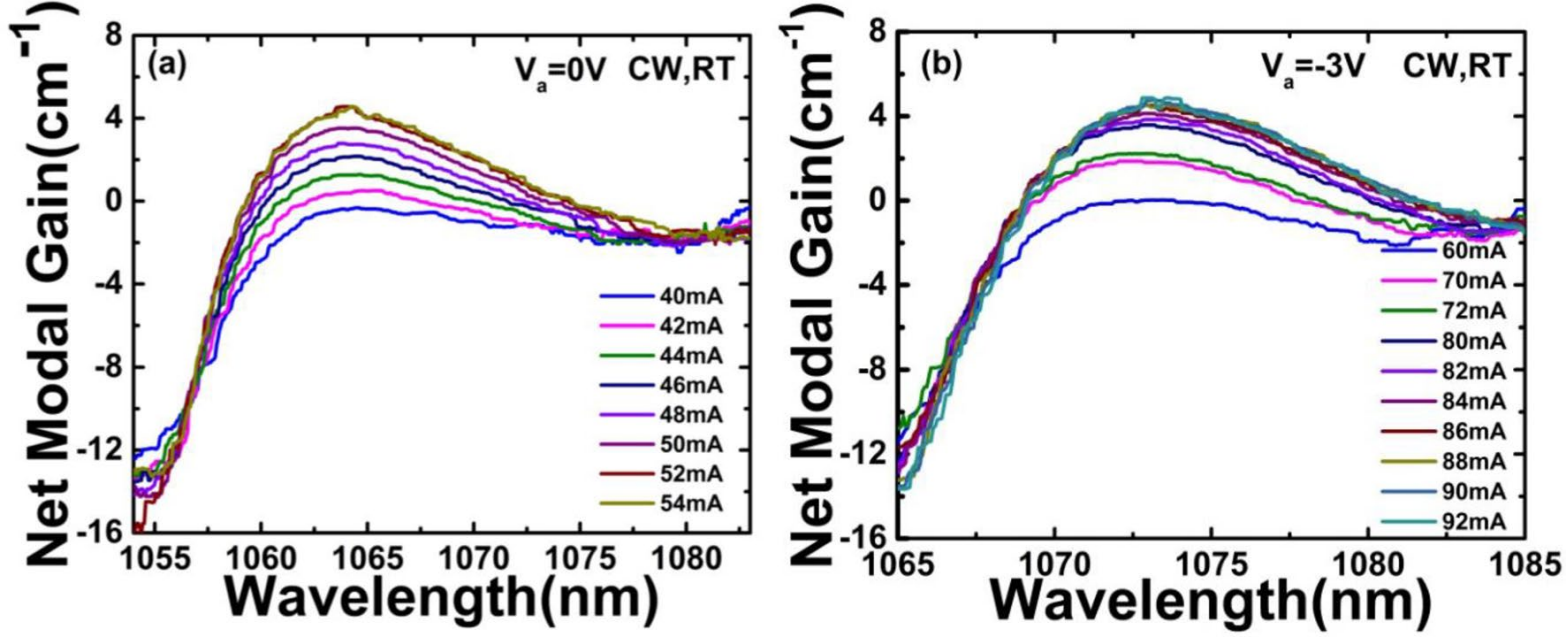
Net modal gain spectra of InGaAs/GaAs DQW lasers at bias *V*_*a*_ (**a** *V*_*a*_ = 0 V; **b** *V*_*a*_ =  − 3 V).

Gain can be determined by amplified spontaneous emission (ASE) spectrum measurement. The net modal gain (*G*_net_) of the two-section MLL was computed using the Hakki-Paoli method. Figure 7 shows the net modal gain spectra at different *I*_g_ when *V*_a_ was set at 0 and − 3 V, respectively. The net modal gain is extracted from the peak to valley ratio of sub-threshold Fabry–Perot oscillations, which was computed using the following relation^21^.

1 $$G_{net} (\lambda ) = \Gamma g_{m} (\lambda ) - \alpha_{i} = \frac{1}{L}\ln \frac{{\sqrt {S(\lambda )} - 1}}{{\sqrt {S(\lambda )} + 1}} + \frac{1}{2L}\ln \left( {\frac{1}{{R_{1} R_{2} }}} \right)$$

where *Γ* is the confinement factor, *g*_m_ is the material gain, *α*_i_ is the internal loss related to defects and dopants in the waveguide and QW. *S* is the peak-to-valley ratio of the FP resonances which can be directly obtained from the ASE spectra, *L* is the whole cavity length (~ 1.99 mm), and *R*_1_, *R*_2_ are facet reflectivity (A value of 0.346 is used for both cleaved facets). The ASE spectra were recorded with a wavelength step size of 0.02 nm. From Fig. 7, it can be seen that, the net modal gain increases with increasing current, and reaches a value close to the mirror loss (α_m_ = (1/2*L*)*ln*(1/*R*_1_*R*_2_) =  ~ 5.33 cm^−1^), which is the threshold loss.

In the following, a comparison of ASE and gain spectra for InGaAs/GaAs DQW MLLs is implemented. Figure 7 plots the modal gain spectrum as a function of gain section currents, which is derived from the ASE spectra as shown in Fig. 6, and it also shows the lasing spectrum near threshold. The laser lases exactly at wavelength of the net modal gain peak, and same results are observed at other *V*_*a*_, including an internal loss, *a*_*i*_ = ~ (1.56 ± 0.34) cm^−1^. An obvious redshift in the gain peak is also observed with increasing *V*_a_, which is attributed to carrier thermalisation within the DQW active layer. It can be seen that the distribution of net modal gain curve tends to be closer near the threshold current at a larger negative *V*_a_ value. From formula (1), a larger negative *V*_a_ causes the first item on the right side of the formula to become closer to zero, which means the value of *S* approaching to + ∞.When *S* value is getting larger, the modes of the MLL becomes more stable, also exhibiting a higher power output.

**Figure 8:**
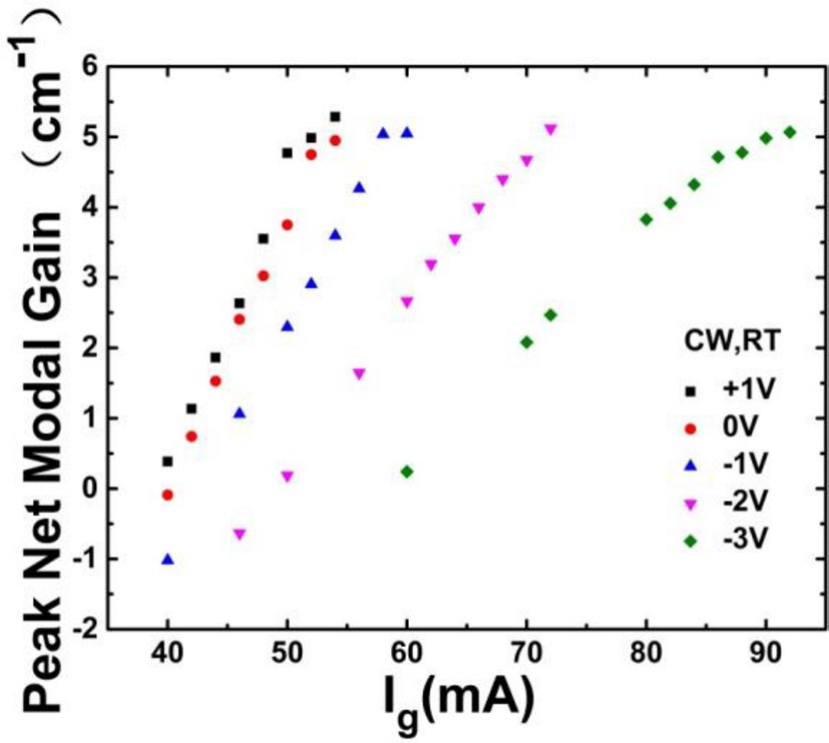
Peak net modal gain versus gain section current under t + 1 V, 0 V, − 1 V, − 2 V, and − 3 V, respectively.

Figure 8 shows the peak net modal gain as a function of gain section injection current at + 1, 0, − 1, − 2 and − 3 V bias voltage, respectively, all measured just at or below the threshold. With increasing reverse bias voltage, the peak net modal gain decreases at constant gain current, which revealed that the dissociation time of the exciton (the width of exciton absorption peak) in the saturable absorber determines the pulse width of mode locked laser. The exciton binding energy of InP system is higher than that of InGaAs/GaAs, and the latter is higher than that of GaAs/AlGaAs^27^. The exciton absorption peak shift toward the longer wavelength as the reverse bias is applied (QCSE effect). On the other hand, the gain peak will shift slightly toward shorter wavelength due to band filling effect as the cavity loss increases with reverse bias voltage. Finally, the large the reverse bias voltage is always together with the more obvious the red shift of the peak wavelength, indicating that the thermal effect of the DQW region is also stronger. As a result, the gain peak and absorption peak are separated (as Refs.^28,29^ also observed). Therefore, in this paper, the bandgap of SA was shifted to the shorter wavelength to match the gain and the absorption peaks. This is benefits to generate short pulse for mode-locked lasers. Peak gain saturation trend is also observed under a high bias voltage at room temperature. The slope of peak net modal gain versus current declines obviously with reverse bias voltage increasing, which should be caused by the increased the thermal effect of photocurrent in the absorber^30,31^. This will be further discussed in the following part.

**Figure 9:**
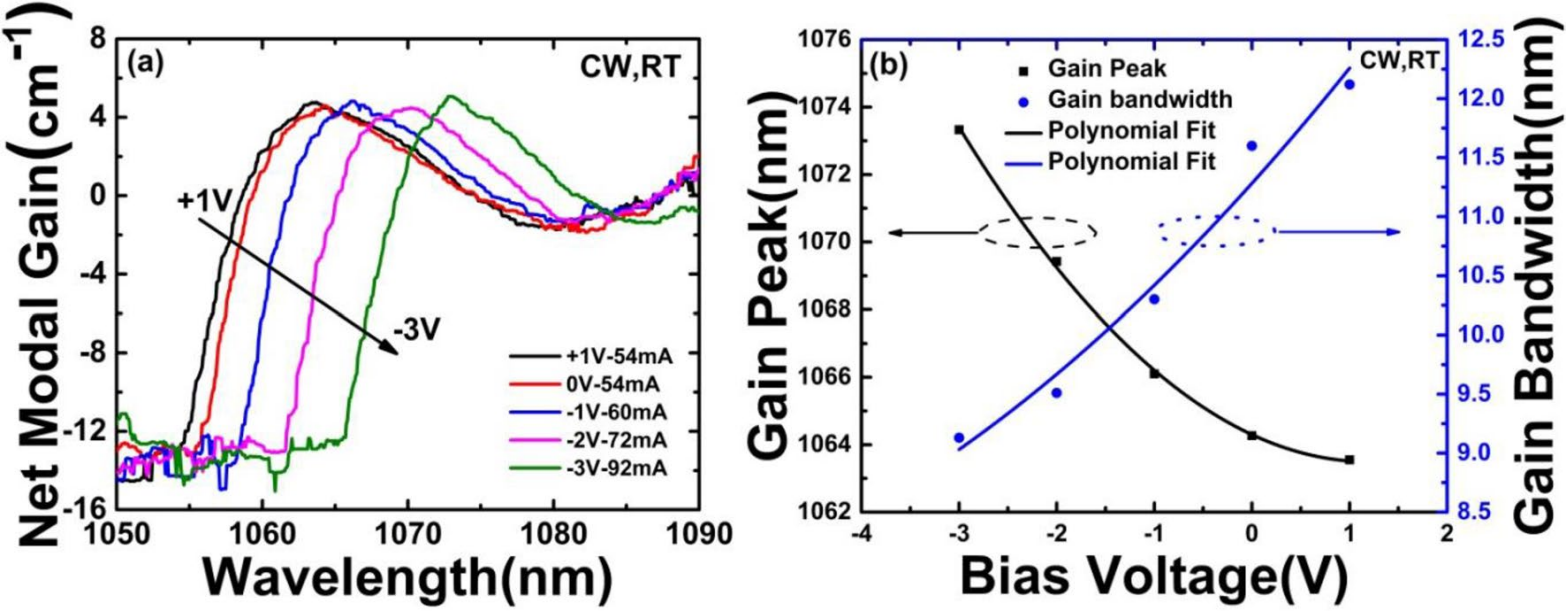
(**a**) *V*_*a*_-dependent threshold gain spectra of the MLL from + 1 to − 3 V. (**b**) Modal gain peak (Black square and polynomial fitting line: y = (1064.3009 ± 0.09679) + (− 1.34671 ± 0.09863)x + (0.56214 ± 0.04245)x^2^, Residual Sum of Squares = 0.05045, R^2^ (COD) = 0.99923, Adj.R^2^ = 0.99846) and gain bandwidth (blue circle and polynomial fitting line: y = (11.28257 ± 0.17727) + (0.91986 ± 0.18065)x + (0.05643 ± 0.07774)x^2^, Residual Sum of Squares = 0.16921, R^2^ (COD) = 0.97484, Adj. R^2^ = 0.94969) of the MLL versus bias voltage, respectively.

In Fig. 9, the net modal gain spectra at threshold for the same sample at different *V*_a_ are plotted. The linear fitting is usually used to derive the internal loss (*α*_i_), which relies on the assumption that the internal differential efficiency keeps constant for lasers with different cavity length. The modal internal loss (*α*_i_) of the DQW laser is derived to be (1.56 ± 0.34) cm^−1^, which is a higher value than the data reported previously^32^. The high internal loss should be caused by the significant shrinking of band gap of the saturated absorption region under negative *V*_a_. The *V*_a_-dependent net modal gain spectra from + 1 to − 3 V, are measured near threshold current. It can also be seen from Fig. 9 that the gain peak is red-shifted and the gain bandwidth is narrowed. These factors lead to an increase in internal optical loss. This is consistent with the internal loss result of the test. At first, we assumed that the nonlinear change is closer to reality. Therefore, a nonlinear fit was chosen in the Fig. 9b. Finally, the agreement between the fitting and the experimental results also confirms our guess.

The net modal gain peak redshifts consistently about 10 nm with increasing negative *V*_a_, which may be mainly due to the absorption spectrum shift by the quantum well band-gap shrinking effect. Since the lasing start exactly at the location of the net modal gain peak of the MLL, it also implies a potential application for wavelength tunable or multi-wavelength laser^33^. Both of the polynomial fitting curves imply a very good adjustability. As expected, at all absorber biases, the peak value of net modal gain has nearly a similar value which is mainly determined by mirror loss.

**Figure 10:**
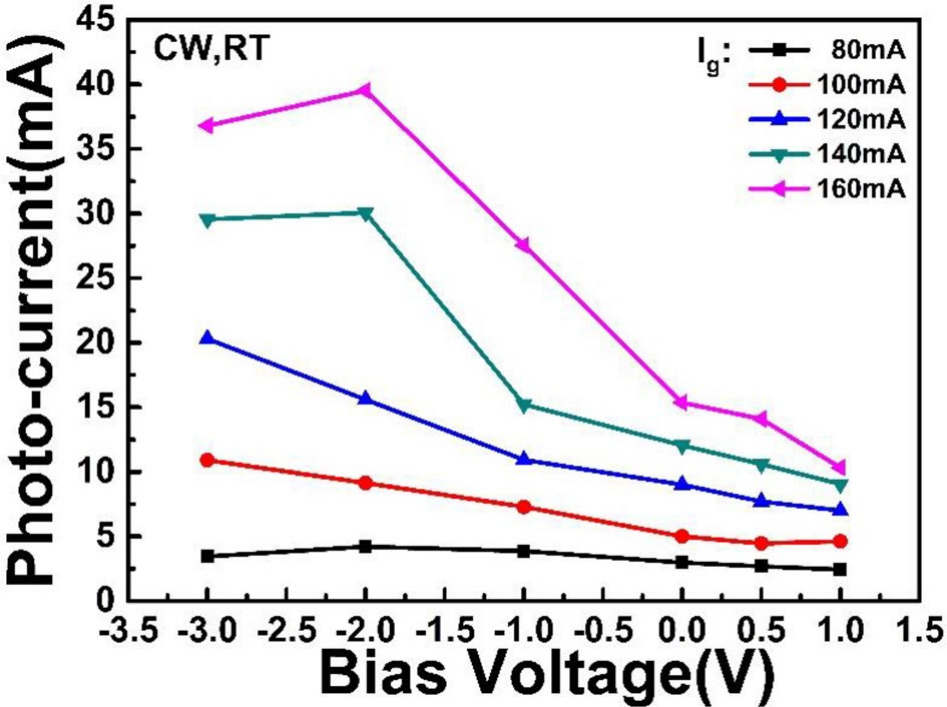
Photocurrent versus bias voltage under different *I*_g_.

Figure 10 shows the photocurrent (*I*_s_) at absorption region increases as *I*_g_ increases, and increases with the *V*_*a*_ negatively increasing. *I*_s_ comes from the saturated absorption region under reverse bias, and its current direction and magnitude are related to the applied bias. When *I*_g_ > 140 mA, *I*_s_ saturates in the reverse bias voltage range of − 3 to − 2 V. Comparing the effect of *I*_g_ on photocurrent, a higher *I*_g_ has much greater influence on the photocurrent of MLL, while *V*_a_ has important effect on photocurrent saturation. For example, when *V*_a_ is in the range of − 3 to − 2 V, *I*_s_ increases with *V*_a_ negatively increasing, and then saturates. Especially, in Fig. 10, the increment of *I*_s_ versus gain current (*I*_*g*_) is uneven under − 2 V bias. And the efficiency that gain current converts photon into photocurrent gets to the highest value from 120 to 140 mA under − 2 V reverse bias, which indicates the most photons are absorbed by the absorption section. So mode-locking always happen at high photon absorption status of the absorber, such as *V*_a_ =  − 0.9 V while *I*_g_ = 180 mA, as shown in Fig. 4a.

We believe that the influence is not a linear process, but probably a nonlinear process. With reference to Fig. 10, it can be seen that the increase of the injection current in the gain region leads to the increase of the photocurrent generated in the reverse bias region, which is not always proportional when the reverse bias voltage is constant. This non-linear relationship necessarily results in a non-linear effect on the pulse width. At the same time, as the reverse bias voltage increases, there is always a saturation photocurrent value. After this maximum value of photocurrent, even if the reverse bias voltage continues to increase, most of the photocurrent values will decrease, and the corresponding optical pulse width will also change accordingly. The pulse width varies with the bias voltage and injection current, corresponding to have an optimal value.

## Conclusion

A monolithic two-section InGaAs/GaAs DQW MLL with asymmetric waveguide emitting at ~ 1.06 μm is demonstrated, and its mode-locking characteristics have been investigated. The mechanisms of the operating behavior are analyzed based on the gain and the saturable absorption in the device.

The fundamental repetition rate with more than 42 dB signal to noise ratio (SNR) is measured at ~ 19.56 GHz, and an ultra-short pulse width (PW) of 1.1 ps is achieved with a peak power of ~ 3.3 W. These results are generally better than the reported by most traditional electrically pumped edge emitting semiconductor MLL. The spectral width and peaks are measured, which become narrower and higher as the bias *V*_a_ is negative increased, and the MLL is redshifted while operating in the above changing. The gain peak redshift is observed with increasing *V*_a_, which may be attributed to carrier and photocurrent thermalisation in the DQW active layer. The net modal gain spectrum of MLL with InGaAs DQW has been measured under different bias *V*_a_ and at gain region current *I*_g_. An internal loss of (1.56 ± 0.34) cm^−1^ is achieved. It also is found that the photocurrent tends to saturate under high reverse bias voltage. And the PW and peak power can still be further optimized by the multi-section or other structure designs of MLL.

Overall, the results show monolithic two-section InGaAs/GaAs DQW MLL with asymmetric waveguide is more suitable for generating shorter light pulses, and there are of great significance to the research of ultrafast semiconductor mode-locked lasers.
